# Valorisation of the invasive alga *Rugulopteryx okamurae* through the production of monomeric sugars

**DOI:** 10.1007/s00253-023-12402-w

**Published:** 2023-02-03

**Authors:** Cristina Agabo-García, Luis I. Romero-García, Carlos J. Álvarez-Gallego, Ana Blandino

**Affiliations:** Faculty of Science, Department of Chemical Engineering and Food Technology, Wine and Agri-Food Research Institute (IVAGRO) and International Campus of Excellence (ceiA3), University of Cadiz, Campus de Puerto Real, s/n. 11510, Puerto Real, Cádiz, Spain

**Keywords:** Invasive brown macroalgae, *Rugulopteryx okamurae*, Biological pretreatment, Solid-state fermentation, *Aspergillus awamori*, Enzymatic hydrolysis

## Abstract

**Abstract:**

*Rugulopteryx okamurae* is an invasive brown alga causing severe environmental and economic problems on the western Mediterranean coasts. Thus, in addition to the difficulties caused to the fishing and tourism sectors, there is a need to manage its accumulation on the beaches. This work aims to valorise this waste by using it as raw material for producing monosaccharides through a two-stage sequential process. These sugars could be used for different fermentative processes to obtain high-value-added bioproducts. In this work, biological pretreatment of the previously conditioned seaweed with the fungus *Aspergillus awamori* in solid-state fermentation (SSF), followed by enzymatic hydrolysis with a commercial enzyme cocktail, was performed. The effect of the extension of the biological pretreatment (2, 5, 8 and 12 days) on the subsequent release of total reducing sugars (TRS) in the enzymatic hydrolysis stage was studied. To analyse this effect, experimental data of TRS produced along the hydrolysis were fitted to simple first-order kinetics. Also, the secretion of cellulase and alginate lyase by the fungus, along with the biological pretreatment, was determined. The results suggest that 5 days of biological pretreatment of the macroalgae with *A. awamori* followed by enzymatic saccharification for 24 h with Cellic CTec2® (112 FP units/g of dry biomass) are the best conditions tested, allowing the production of around 240 g of TRS per kg of dried biomass. The main sugars obtained were glucose (95.8 %) and mannitol (1.5 %), followed by galactose (1 %), arabinose (0.9 %) and fucose (0.5 %).

**Key points:**

*• Five-day SSF by A. awamori was the best condition to pretreat R. okamurae.*

*• Five-day SSF was optimal for alginate lyase production (1.63 ±0.011 IU/g biomass).*

*• A maximum yield of 239 mg TRS/g biomass was obtained (with 95.8 % glucose).*

**Graphical Abstract:**

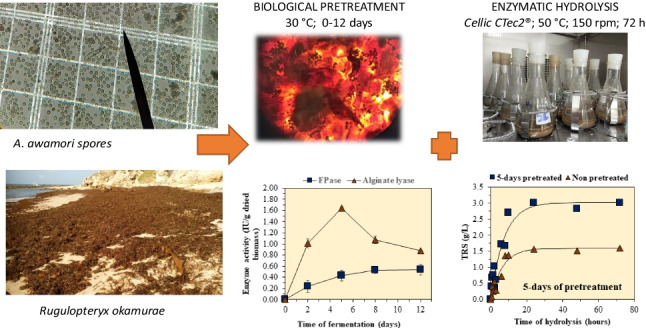

## Introduction

Macroalgae or seaweed are gaining attention in the scientific community as valuable economic and ecological resources for a wide range of biorefining processes. Also, they are used as a source of diverse bioactive compounds to treat multiple diseases such as cancer, diabetes, inflammation, dementia and others and are consumed regularly by people from East Asian countries such as Korea, China and Japan (Rengasamy et al. 2014). This habit has been widespread throughout Europe and America, probably due to the benefits of bioactive compound content in macroalgae (Rengasamy et al. 2020). However, not all species are useful for the culinary or pharmacological sectors. In this sense, *Rugulopteryx okamurae* is a brown macroalga from the family *Dictyotaceae* coming from the Pacific Ocean, normally found off the coasts of Japan, China and Korea. However, this macroalga has recently settled in areas of the European coasts, such as the Strait of Gibraltar, causing severe problems to the natural ecosystem (García-Gómez et al. 2021). This species has been considered invasive by the Spanish Government Royal Decree 630/2013 (2013), and it is a factual problem for the fishery sector in the Gulf of Cádiz (Spain). In fact, towards the end of spring, the coast is usually cleaned to remove massive deposits of the macroalga to prepare it for the tourist season, which implies additional costs for the village councils (García-Gómez et al 2020). For these reasons, numerous investigations are trying to control its expansion and/or looking for different applications of this species as an alternative to its withdrawal to landfills. In this sense, the biorefinery approach could be a promising alternative to generate valuable products such as biomethane (Farghali et al. 2021), phenolic compounds (antioxidants), sugars and proteins (Trigueros et al. 2021) and bioethanol (Rocher et al. 2021), from algal biomass. For brown algae species with a commercial interest, it is usual to valorise the waste generated after polymer extraction (i.e. alginate, fucoidan). Among other options, it has been widely used to produce fermentable sugars to be exploited to obtain high-added-value products, such as organic acids or ethanol (Barbot et al. 2016).

Macroalgae are classified into three major groups: green macroalgae, brown macroalgae and red macroalgae (Hong et al. 2014). Brown and red macroalgae are more actively produced and utilised than green macroalgae, accounting for 76% of the total amount of macroalgae production (Roesijadi et al. 2010). The carbohydrate composition of brown macroalgae is complex and includes the cell-wall polysaccharides, alginate and fucoidan, and the storage polysaccharide laminarin and mannitol (Groisillier et al. 2014). Mannitol is a sugar alcohol form of mannose, and laminarin is a linear polysaccharide of mannitol-containing β-1,3-linked glucose (Ji et al. 2016). Alginate is made from two copolymers, β-l-guluronic acid and α-d-mannuronic acid residues (Kuznetsova et al. 2020). Finally, the polymer fucoidan shows different monosaccharide compositions depending on the species, growth environment or season of harvesting (Chen et al. 2021). Therefore, besides fucose, it may contain other sugars, like xylose, arabinose, rhamnose, glucose or galactose, among others (Citkowska et al. 2019). Brown algae also contain cellulose in small amounts in the cell wall, which is composed of at least two different layers. While the inner layer is mainly made up of cellulose and imparts rigidity to the wall, the outer one is an amorphous matrix composed of alginate and fucoidan and imparts strength and flexibility to the cell wall (Davis et al. 2003). Alginates, fucoidans and cellulose are in an average weight ratio of 3:1:1 in mature intertidal brown algae (Gurvan et al. 2010).

The production of monomeric sugars is a very important issue since a significant number of value-added products can be obtained through fermentation processes. For example, regarding the main sugar monomers derived from brown seaweeds, in addition to glucose, D-galactose, D-mannitol and L-fucose have been classified as fermentable sugars for lactic acid bacteria. However, non-fermentable sugars include D-mannuronic acid and L-guluronic acid. In fact, it has been reported (Hwang et al. 2011) that the yield for lactic acid production from sugars derived from seaweeds is comparable to the corresponding for lignocellulosic waste. The use of recombinant microorganisms for the utilisation of non-fermentable sugars from algae is an exciting option but is still in development.

Given the complex composition of brown macroalgae, an enzyme cocktail with different hydrolytic activities is generally needed for the efficient hydrolysis of these polymers to simple sugars, which can be subsequently fermented to different bioproducts in the framework of a biorefinery approach (Bayu et al. 2021). Indeed, most of the commercially available enzymatic cocktails are tailored for lignocellulosic material and are not adapted for marine biomass. For this reason, those available are extremely expensive, which makes their application on an industrial scale difficult. Therefore, for efficient brown seaweed hydrolysis, the supplementation of the alginate lyase activity is required. For example, Ravanal et al. (2016) tested the pretreatment of the brown macroalga *Macrocystis pyrifera* with 2 % (v/v) H_2_SO_4_ for 1 h and the saccharification with a cocktail of cellulases and alginate lyases, releasing 68.4 % of glucose and 85.8 % of uronic acid from the theoretical monosaccharide content. Later, the same research group saccharified two brown algae species (*Macrocystis pyrifera* and *Saccharina latissima*) by using a sequential enzymatic treatment: firstly, with commercial and isolated alginate lyases from different microorganisms and, secondly, using the commercial pool of cellulase and β-glucosidase Cellic CTec2®. They found that alginate lyases are only helpful for the saccharification of native macroalgae, while the effect of this enzyme is limited in pretreated algae. The highest glucose yield (94.5 %) was reached when native *S. latissima* was saccharified with a mixture of alginate lyases H plus oligoalginate lyases, followed by the addition of Cellic CTec2® (Ravanal et al. 2017).

As it is well known, the enzymatic hydrolysis of algal biomass is usually improved by the application of different pretreatments which facilitate the attack of enzymes to release simple sugars (Thompson et al 2019; Fasahati et al. 2015; Lee et al. 2013). Biological pretreatment of macroalgae by the growth of a proper microorganism in solid-state fermentation (SSF) is a competitive and effective option because it involves a multiple enzyme process (Trivedi et al. 2015). It is also considered a relatively cheap and environmentally friendly pretreatment for improving the biodegradability of macroalgal biomass (Tapia-Tussell et al. 2018). However, only a few studies have evaluated the use of different microorganisms to pretreat macroalgae (Norakma et al. 2022; Yahmed et al. 2017).

In this work, a biorefinery approach is proposed to valorise the invasive macroalga species *R. okamurae* to produce monosaccharides by two-stage sequential process: (i) biological pretreatment with the fungus *Aspergillus awamori* in a solid-state fermentation process and (ii) enzymatic hydrolysis of the pretreated biomass with the commercial enzyme cocktail Cellic CTec2®. It can be emphasised that this is a waste that needs to be managed with the additional advantage of being a cost-free source of substrate for biorefinery processing, contributing to increases in the added value of the products obtained. In addition, this brown alga is present in large quantities in the western Mediterranean littorals all year round, causing severe environmental impact and economic cost. Its recollection and use as raw material for obtaining monomeric sugars to be used in the carbohydrates-based biorefinery could be a suitable strategy for its integration in a circular economy-type proposal. So far, to the best of our knowledge, only one paper describes the use of *A. awamori* for the biological pretreatment of macroalgae (Agabo-Garcia et al. 2022). The capability of *A. awamori* of secreting hydrolytic enzymes, such as xylanases (Gouka et al. 1996), pectinases (Blandino et al. 2002; Botella et al. 2007; Díaz et al. 2016) or hemicellulases (Potanen et al. 1986) among others, when it is grown on different substrates, has been extensively reported in the literature. As detailed by dos Santos Silva et al. (2022), alginate lyase production has been usually performed in submerged fermentation by bacteria. However, filamentous fungi are also capable to produce this enzyme, and some species, such as *Cunninghamella echinulata*, have been tested (dos Santos Silva et al. 2022). As far as we know, we report for the first time in this study the production of alginate lyase by *A. awamori* in SSF. Also, no reference has been found about the use of *R. okamurae* for biorefinery purposes.

## Materials and methods

### Biological materials

#### Substrate sampling and conditioning

The brown macroalga species *R. okamurae* was manually collected from the beach shore at Tarifa (Cádiz, South of Spain) in February–March. Samples were washed with tap water to dilute the concentration of salts and eliminate sand and other impurities. For this purpose, the seaweed was placed in continuously overflowing tanks for 24 h. Then, cleaned algal biomass was dried at a controlled temperature (25–30 °C) for 2 days in a greenhouse to ensure long-term storage. Dried algae were finely grounded with an ultracentrifugal mill (Retsch ® zm 200) at 10,000 rpm. Particle size distribution was in the range of 0.063–1.5 mm.

#### Inoculum for SSF

*Aspergillus awamori* 2B.361 U2/1 (ABM Chemicals®, Woodley, Cheshire), a sequential mutant of *Aspergillus niger* NRRL 3312, was grown in Petri dishes at 30 °C for 5 days using a synthetic medium composed by (g/L) 1 peptone, 0.5 yeast extract, 15 agar, 6 xylan, 5 avicel and 1 pectin. Spores were harvested by lightly scraping the plate with 2 mL sterile water containing 0.9% (w/v) NaCl. The number of spores in the suspension was counted using an improved Neubauer chamber and an optic microscope (Leica® DME). The final spore concentration of the suspension used for the inoculum was 7.25 ·10^8^ spores/mL (Díaz et al. 2012).

### Biological pretreatment

Biological pretreatment of *R. okamurae* was performed by SSF with the fungus *A. awamori*. SSF was carried out in 250-mL Erlenmeyer flasks containing 2 g of conditioned *R. okamurae* moistened with 6 mL of Mandels’ salts solution composed by (g/L) 0.3 urea, 1.4 (NH_4_)_2_SO_4_, 2.0 KH_2_PO_4_, 0.3 CaCl_2_, 0.3 MgSO_4_, 0.25 yeast extract, 0.75 peptone, 0.005 FeSO_4_·7H_2_O, 0.020 CoCl_2_, 0.0016 MnSO_4_ and 0.0014 ZnSO_4_ (Yahmed et al. 2017). Flasks were autoclaved at 121 °C for 20 min, then cooled down and inoculated with the proper volume of the spore suspension to reach 4.4·10^8^ spores per g of dried algal biomass. All the flasks were incubated in static conditions at 30 °C for 12 days, and samples were taken at different times (0, 2, 5, 8 and 12 days). Fermentation conditions were established according to previous studies of the research group (Díaz et al. 2012).

**Fig. 1:**
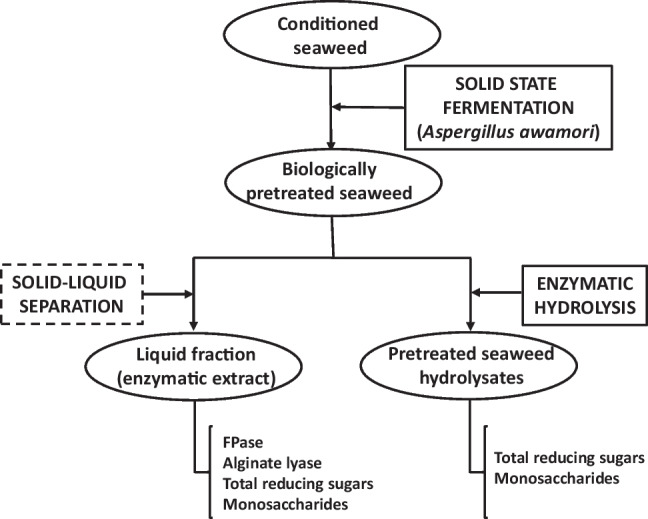
Protocol performed in this study for *R. okamurae* processing

Total reducing sugars (TRS), cellulase (FPase) and alginate lyase activities produced along the SSF were measured in the liquid extracts obtained, as described below. In addition, fermented samples were enzymatically hydrolysed following the protocol detailed later. All fermentation tests were performed in triplicate (see Fig. 1).

### Enzymatic hydrolysis

Two grams of biologically pretreated and also non-pretreated biomass were added to 250-mL Erlenmeyer flasks and suspended in 90 mL phosphate buffer 0.05 mol/L at pH 5. Flasks were sterilised in an autoclave (121 °C for 20 min), and after cooling them down, 344 µL of enzyme cocktail Cellic CTec2® (Novozyme®) (117.46 FPase units/g of biomass) were added. Then, they were incubated at 50 °C and 150 rpm for several days in a rotary shaker (Thermoscientific® MAXQ6000). During the hydrolysis process, samples were taken periodically (2, 5, 8 and 12 days) and centrifuged at 10,000 rpm for 10 min (Eppendorf® 5810 R), removing the pellet and collecting the supernatant for the analysis of TRS and the monosaccharide profile. All hydrolysis assays were made in triplicate.

### Enzyme extraction and activities

Fermented *R. okamurae* contained in 250-mL Erlenmeyer flasks (2 g) was suspended in 20 mL of 0.1 % v/v Tween 80 and incubated in a rotary shaker (MAXQ6000, Thermoscientific®) for 30 min at 4 °C and 150 rpm. Then, suspensions were centrifuged (Eppendorf 5810R) at 10,000 rpm and 4 °C for 10 min, and the supernatant liquor (the enzymatic extract) was used to measure the cellulase (FPase, EC 3.2.1.91) and the alginate lyase (EC 4.2.99.4) activities as well as the total reducing sugar concentration (TRS) and the monosaccharide profile.

FPase activity was measured by incubating 0.5 mL of the diluted enzymatic extract with 1 mL of citrate buffer (0.05 M, pH 4.8), containing a Whatman® No.1 filter paper strip (1 × 6 cm, 50 mg), at 50 °C for 60 min. Then, the TRS produced were measured by the DNS method adapted to microplate.

Alginate lyase activity was measured by incubating 0.2 mL of the enzymatic extract with 1.8 mL of sodium alginate 8 % w/v in citrate buffer 0.05 M at pH 6.3 and 30 °C for 90 min. After that, 2 mL of dinitrosalicylic acid solution were added, and the tubes were incubated at 105 °C for 10 min. After cooling them down, 50 µL of the sample were diluted with 250 µL of deionised water and transferred to a microplate for TRS measurement by the DNS method.

One unit of FPase/alginate lyase activity was defined as the amount of enzyme required to release 1 μmol of reducing sugars per minute at defined assay conditions.

### Analysis methods

#### Conductivity and ion analysis

Conductivity and ion concentrations were measured in the leachate obtained by disgregation of 1g of the conditioned macroalgae with 100 mL of distilled water for 30 min. Conductivity was measured by using a conductivity metre type (WTW® LF 330). The concentrations of anions were determined by ion chromatography (940 Professional IC Vario, Metrohm). The cationic content was determined by Ion Metrohm 930 Compact IC flex.

#### Sugar analysis

TRS were measured by the dinitrosalicylic acid method (DNS) adapted to a microplate (Gonçalves et al. 2010) by using a microplate reader type Epoch® 12. Monosaccharides were measured by ion chromatography (Metrohm 930 Compact IC Flex) with a pulsed amperometric detector with a gold electrode. Elution was carried out in isocratic at a 0.5 mL/min flow rate with 300 mM NaOH and 1 mM NaOAc. Separation was achieved on a Metrosep Carb 2- 150/4.0 column (Metrohm).

Fucose was measured by an enzymatic kit (K-fucose 05/20) from Megazyme® adapted to a microplate. By this kit, L-fucose is oxidised by the enzyme L-fucose dehydrogenase in the presence of nicotinamide-adenine dinucleotide phosphate (NADP+) to L-fucono-1,5-lactone with the formation of reduced nicotinamide-adenine dinucleotide phosphate (NADPH) (Morris 1988).

#### Fibre analysis

Detergent fibre analysis of biologically pretreated and no-pretreated *R. okamurae* was carried out using Fibertec^TM^ 8000 following the method of Van Soest and McQueen (1973). Acid detergent fibre (ADF) and acid detergent lignin (ADL) analysis were performed according to EN ISO 13906:2008, while amylase-treated neutral detergent fibre (aNDF) analysis was applied according to AOAC 2002:04/ISO 16472:2006. These methods allow the quantification of different fractions: (i) fraction of compounds extracted with acetone detergent (AD) which correspond to pigments and lipids and (ii) fraction removable with neutral detergent (NDF) which includes compounds such as alginates, laminarin, fucoidan, proteins and polyphenolic compounds. In this fraction, it also can be distinguished between removable non-calcined (soluble salts) and removable calcined (rest of extractable material but not saline), (iii) removable with acid detergent (ADF) and extractable with concentrated acid (ADL) which contain Klason lignin, cellulose and residual polysaccharides (Möller 2005, 2008).

### Glucose yield

The overall glucose yield of whole processing of the seaweed (combination of pretreatment and enzymatic hydrolysis) was calculated according to the typical expression for the yield of cellulose hydrolysis (Spano et al. 1976) adapted by Betlej et al. (2022):

$$\begin{pmatrix}Glucose\\yield\end{pmatrix}\;\left(\%\right)=\frac{\begin{pmatrix}glucose\;concentration\\in\;hydrolysate\end{pmatrix}\left(\frac{g\;glucose}{g\;dry\;algal}\right)}{1.11\begin{pmatrix}cellulose\;concentration\\in\;the\;seaweed\end{pmatrix}\left(\frac{g\;glucose}{g\;dry\;algal}\right)}\cdot100$$

### Kinetic model

To analyse the effect of the biological pretreatment with *A. awamori* of the conditioned algal biomass on its enzymatic hydrolysis rate, the experimental data of TRS produced along the hydrolysis were fitted to simple first-order kinetics:

$$\left(-\frac{\mathrm{dS}}{\mathrm{dt}}\right)=\mathrm{k}\cdot \mathrm{S}$$

where *S* is the concentration of substrate (hydrolysable polysaccharides), *t* is the pretreatment time and *k* corresponds to the first-order kinetic constant.

Considering a direct relationship between the amount of hydrolysed substrate and the product obtained P (total reducing sugars released), the following equation could be used:

$$\frac{\mathrm{dP}}{\mathrm{dt}}=\upbeta \cdot \left(-\frac{\mathrm{dS}}{\mathrm{dt}}\right)$$

where *β* is the constant which represents the substrate-to-product yield coefficient.

Assuming that *P* = 0 at *t* = 0, the integration of the above equation gives the following mathematical expressions:

$$\mathrm{P}=\upbeta \cdot \left({\mathrm{S}}_{\mathrm{o}}-\mathrm{S}\right)\Rightarrow \mathrm{S}={\mathrm{S}}_{\mathrm{o}}- \frac{P}{\beta }$$

And therefore:

$$\left(-\frac{\mathrm{dS}}{\mathrm{dt}}\right)=\frac{1}{\upbeta }\cdot \left(\frac{\mathrm{dP}}{\mathrm{dt}}\right)=\mathrm{k}\cdot \mathrm{S}=\mathrm{k}\cdot \left[{\mathrm{S}}_{\mathrm{o}}-\frac{P}{\beta }\right] \Rightarrow \frac{\mathrm{dP}}{\mathrm{dt}}=\mathrm{k}\cdot [\upbeta \cdot {\mathrm{S}}_{\mathrm{o}}-\mathrm{P}]$$

The integration of the above equation gives:

$$\mathrm{P}=\upbeta \cdot {\mathrm{S}}_{\mathrm{o}}\left(1-{\mathrm{e}}^{-\mathrm{kt}}\right)={P}_{max}\left(1-{\mathrm{e}}^{-\mathrm{kt}}\right)$$

where,

*P*: TRS concentration (g/L) at any time t
*P*_max_: Theoretical maximum value of TRS which can be obtained (g/L)
*K*: Hydrolysis rate constant (1/h)
*T*: Time (h)

To calculate the values of the parameters *P*_max_ and *k*, the first-order kinetic model was fitted to experimental data of TRS released with hydrolysis time by using the non-linear regression method provided by the Statgraphics© Centurion 18 software.

## Results

### Algal biomass characterisation

The algal biomass chemical composition is included in Table 1. As can be seen, *R. okamurae* has a low content, about 4.5 %, in compounds removable with acetone such as lipids and pigments. The fraction removable with neutral detergent (NDF) which includes compounds such as alginates, laminarin, fucoidan, proteins and polyphenolic compounds accounts for about 68 %, while removable with acid detergent (ADF) and extractable with concentrated acid (ADL) which contains Klason lignin, cellulose and residual polysaccharides only represent 27.8 %. From the NDF fraction, only 14.8 % was found in form of salts.

**Table 1:** *Rugulopteryx okamurae* chemical composition

Solid characterisation	Value (%)
Total solids (TS)^a^	86.8 ± 2.2
Inorganic matter^a^	10.9 ± 1.1
Organic matter^a^	75.9 ± 1.1
Fibre analysis	
Removable with acetone	4.46 ± 0.26
Removable with acid detergent (ADF)	14.0 ± 1.4
Removable with acid concentrated detergent (ADL)	13.9 ± 1.2
Removable with neutral detergent (NDF)	67.7 ± 1.5
In form of salts	14.8 ± 0.0
Free forms	52.9 ± 1.5
Leachate characterisation	**Value (%)**
Conductivity	1.85^b^
Fluoride	N.D.^c^
Chlorides	105
Nitrites	N.D.^c^
Bromide	2.61
Nitrates	4.95
Phosphates	18.9
Sulphates	122
Sodium	57.5
Ammonium	5.12
Potassium	13.9
Magnesium	N.D.^c^
Calcium	N.D.^c^

^a^On dry matter basis

^b^EC units: mS/cm

^c^Not detected

Although alginate is generally found in the cell walls and the intercellular matrix forming insoluble salts with calcium (Ca), magnesium (Mg) or sodium (Na), only sodium cations were detected in the lixiviate.

This salinity of the leachate was measured to assess the efficiency of the washing stage with tap water, obtaining a conductivity of 1.85 mS/cm. Taking into account that the initial conductivity was about 50 mS/cm, a reduction of the salt content upper than 95% was achieved. The chloride ion concentration in the leachate was 105 mg/L, and it is not sufficiently high to affect the fermentation of *R. okamurae* by *A. awamori*.

### Biological pretreatment of algal biomass

**Fig. 2:**
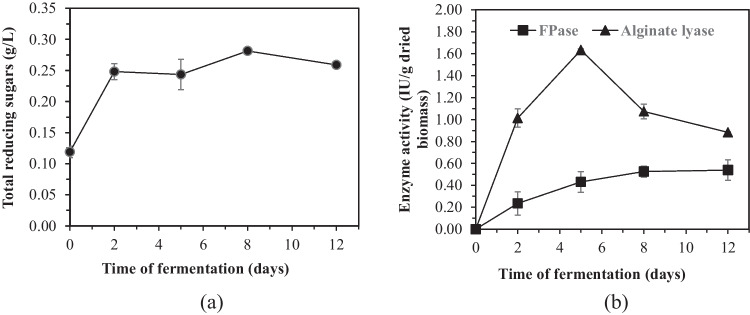
**a** Total reducing sugars and **b** enzymatic activities obtained along the SSF with *A. awamori* of *R. okamurae*

**Fig. 3:**
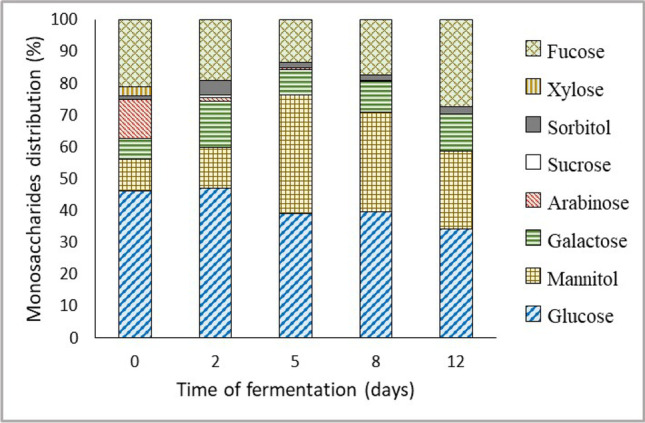
Main monosaccharide distribution along the SSF

As stated above, biological pretreatment of *R. okamurae* was performed by the fungal growth of *A. awamori* in solid-state fermentation (SSF). The evolutions of TRS and the FPase and alginate lyase activities (Fig. 2) with the operation time, as well as the monosaccharide profile (Fig. 3), were monitored to obtain a deeper understanding of the effect of fungal growth on the composition of the hydrolysates from macroalgae.

As can be seen in Fig. 2a, TRS increases sharply after 2 days of SSF, from 0.12 to 0.25 g/L, and then, it starts to increase smoothly to 0.26 g/L. The TRS measured at day 0 of fermentation were generated as a consequence of the autoclaving of the algal biomass. Up to the 2nd day of fermentation, the sugar released rate by the secreted enzymes surpasses the rate of sugar consumption for fungal growth. FPase and alginate lyase profiles are shown in Fig. 2b. As can be seen, while FPase raises smoothly until the 8th day of fermentation, alginate lyase activity increases significantly after 2 days of SSF and peaks on the fifth day. Therefore, the quick increase in TRS after 2 days of fermentation is the result of the combined activity of both enzymes, being probably the effect of alginate lyase more acute. From the 2nd to the 5th day of SSF, the activity of both enzymes increases, while TRS remains almost constant, indicating that the fungus consumes actively the sugars released from the hydrolysis of the polysaccharides. From the 5th to the 8th day, although alginate lyase decreases noticeably, as FPase increases slightly, the TRS concentration remains almost stable. However, from the 8th day, as FPase activity only stabilises and alginate lyase decreases further, the TRS decreases, indicating that the rate of fungal sugar consumption is higher than the enzymatic hydrolysis rate.

The distribution of the main monosaccharides along the SSF is shown in Fig. 3. As was expected, the major sugars in the extracts are glucose, mannitol and fucose. At the beginning of SSF, some monosaccharides were measured, with glucose accounting for around 46.4 % (44.6 ppm), followed by 21.0 % fucose (20.2 ppm), 12.3% arabinose (11.9 ppm), 9.9 % mannitol (9.5 ppm), 6.4 % galactose (6.1 ppm) and 2.8 % xylose (2.7 ppm). As long as fermentation progresses, the relative percentage of mannitol increases reaching 37.3 % (129.5 ppm) after 5 days, while glucose accounts for 39.0 % (135.3 ppm). Regarding fucose concentration, it increased linearly with the days of fermentation, reaching a concentration of 79.14 ppm after 12 days of fermentation. However, in terms of relative percentages, the minimum contribution was calculated after 5 days of pretreatment, with 13.5 % (46.8 ppm). On the contrary, the content of both arabinose and xylose decreases along with the fermentation. Thus, for the 5th day, the first one accounted only 0.5 % (1.8 ppm), and the second one was negligible. Galactose increases until the 8th day, from 6.11 ppm (6.4 %) to 33.7 ppm (9.6 %) and afterwards stabilises, being the fourth major monosaccharide in the extracts. Sorbitol, which is a mannitol isomer, is present in all the extracts in small percentages. Sucrose was only detected in small concentrations on days 0 and 2.

The different monosaccharides in the extracts during the fermentation are coming from the hydrolysis of the constituent polysaccharides of *R. okamurae*. Thus, laminarin generates mannitol and glucose, although this last one can be also released from cellulose and fucoidan. Fucose and the other minor sugars measured in the extract, like arabinose, xylose and galactose, come just from fucoidan so its degradation can be followed by the concentrations of its monomers.

### Enzymatic hydrolysis of algal biomass

**Fig. 4:**
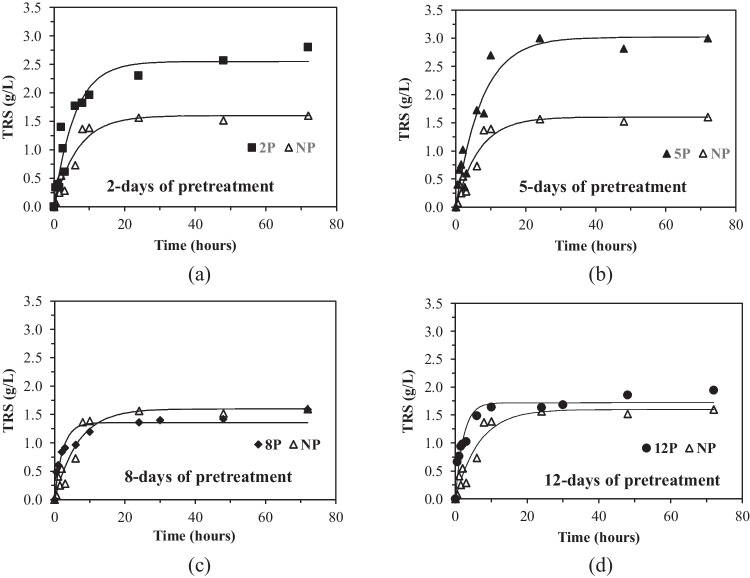
Total reducing sugars (TRS) released along hydrolysis with commercial cocktail Cellic CTec2® for biologically pretreated seaweed for different times **a** 2 days, **b** 5 days, **c** 8 days and **d** 12 days in comparison with non-pretreated (NP) algal biomass

Biologically pretreated algal biomass for 2, 5, 8 and 12 days (2P, 5P, 8P and 12P, respectively) and non-pretreated algal biomass (NP) were hydrolysed with the enzyme cocktail Cellic CTec2®. Experimental data along with predicted trends by the first-order model are shown in Fig. 4. Also, the parameters obtained from fitting the first-order kinetic model to experimental data of reducing sugar concentrations measured in the hydrolysates are shown in Table 2.

**Table 2:** Hydrolysis rate constant (k) and theoretical maximum value of TRS (P_max_) from the enzymatic hydrolysis of non-pretreated seaweed (NP) and biologically pretreated seaweed for 2 days (2P), 5 days (5P), 8 days (8P) or 12 days (12P)

Kinetic parameter	NP	2P	5P	8P	12P
k (1/h)	0.151	0.171	0.134	0.441	0.450
P_max_ (g/L)	1.600	2.550	3.022	1.355	1.722
R^2^	0.9348	0.9310	0.9282	0.8971	0.9236

As can be observed in Fig. 4, the biological pretreatment of the algal biomass for 2 or 5 days has a clear positive effect on the total reducing sugars released in the hydrolysates. *P*_max_ increases from 1.6 g/L for non-pretreated samples to almost a double value (3.0 g/L) for the 5-day-pretreated ones (see Table 2). Concerning the hydrolysis constant k, it seems that the pretreatment for 2 or 5 days does not affect significantly the value of this parameter, as it is slightly higher for the 2-day-pretreated algal biomass but a little bit lower when the pretreatment increases to 5 days. On the contrary, k increases significantly when the biological pretreatment is prolonged to 8 or 12 days. In those cases, the maximum reducing sugar concentration can be achieved around after 7 h of hydrolysis. However, if *P*_max_ values for 8 and 12 days of pretreatment are compared to the corresponding one for non-pretreated algal biomass, only a slight increase for 12-day-pretreated samples can be observed.

Attending to this, we can conclude that 5 days of biological pretreatment is recommended for obtaining a high concentration of TRS in the algal hydrolysates, although the results for 2 days of pretreatment are only slightly lower in terms of TRS. Therefore, although it was not tested, it is possible that maximum TRS concentrations could be achieved for 3 or 4 days of fermentation. Increasing the pretreatment time to 8 or more days has a negative effect on the final sugar concentration of the hydrolysates even when the kinetic effect is positive. The values of the hydrolysis rate constant k for non-pretreated and 2-day and 5-day pretreated seaweed are of the same order of magnitude, and the same happens for algal biomass pretreated for 8 and 12 days. This behaviour may be the result of the fact that the availability of polysaccharides for the enzymatic attack is affected by the duration of biological pretreatment. Thus, for algal biomass pretreated for 8 days or more, the polysaccharides remaining after fungal growth are more readily hydrolysable than those available for pretreatment times of up to 5 days. In this sense, the fungus secretes hydrolytic enzymes which release the sugars needed for its growth, and as the fermentation time increases, the percentage of hydrolysed polysaccharides also does.

### Complete algal biomass processing

Table 3 includes the total reducing sugars produced after the complete algal biomass processing: biological pretreatment (at different duration times) plus enzymatic hydrolysis for 24 h. Those sugars include the ones released during the biological pretreatment stage, in which the final concentration of TRS in the medium is the result of a balance between the sugars released from the activity of hydrolytic enzymes secreted by the fungus and the sugar consumption needed for the fungal growth. In addition, the final total reducing sugar concentration in Table 3 also includes the sugars produced after the hydrolysis stage. It should be noted that, even for non-pretreated algal biomass, a certain amount of reducing sugars is present in the samples before the enzymatic hydrolysis stage, as a consequence of the thermal treatment related to the autoclaving of the algal biomass.

**Table 3:** TRS produced after complete algal biomass processing: biological pretreatment for 5 days (5P), 8 days (8P) or 12 days (12P) followed by enzymatic hydrolysis for 24 h. *N.P* non-pretreated seaweed

Sample	TRS (g/L)	Glucose (%)	Mannitol (%)	Galactose (%)	Arabinose (%)	Fucose (%)
12P	3.62	95.82	1.30	1.20	0.90	0.14
8P	3.40	96.73	1.10	1.14	0.89	0.14
5P	5.32	95.76	1.47	1.02	0.90	0.47
NP	4.27	98.04	0.02	1.21	-	0.20

As can be seen in Table 3, the biological pretreatment of the algal biomass for 5 days increases the TRS concentration by about 25 % compared to the non-pretreated one. However, again a negative impact of the pretreatment time for 8 or 12 days on TRS was observed, with a decrease of 19 % and 15 %, respectively, compared to non-pretreated biomass. The percentages of the principal sugars measured in the samples have also been included. The main monosaccharide in the hydrolysates is glucose, accounting for more than 95 % in all cases. Hydrolysates from non-pretreated algal biomass showed the highest percentage of glucose (98 %), while only a small percentage of mannitol is present. On the contrary, all pretreated samples have a slightly lower percentage of glucose, but they also include in their composition other sugars such as mannitol, arabinose or fucose. The highest concentrations of glucose (5098 ppm), fucose (445 ppm), mannitol (78 ppm), galactose (54 ppm) and arabinose (48 ppm) were measured for 5-day pretreated samples. Considering data for the hydrolysates from 5 days of pretreatment as 100 %, glucose in the hydrolysates for 8-day and 12-day pretreated samples account for 65 and 68 %, respectively, whereas for non-pretreated samples, it represents about 80 %. However, in the case of galactose, the percentage decreases from 100 (5-day pretreated samples) to 94.5 % (non-pretreated samples) and 72.6 and 79.5 %, for 8-day and 12-day pretreated samples, respectively. For arabinose, the percentages are reduced from 100% after 5 days of pretreatment to 64.4 % and 67.8 %, respectively, when time rises to 8 and 12 days, and it was non-detectable in the case of the non-pretreated sample. Regarding fucose, the non-pretreated sample contained 43.3 % of this sugar, and after 8 days and 12 days of pretreatment, its content was reduced to 29.7 % and 29.1 %, respectively.

Therefore, maximum TRS concentration was obtained for samples pretreated for 5 days, with a TRS yield of 239 g/kg of dry biomass, which corresponds to a glucose yield of about 230 g/kg of dry biomass.

The glucose yield was calculated as detailed in the “Materials and Methods” section. The cellulose concentration in the seaweed in the equation was first considered the ADL fraction from the fibre analysis. However, the glucose yields obtained for the different pretreatment times are over 100 % (ranging from 96 to 149 %). This is because other polymers different from cellulose can be present in the ADF fraction, including hemicellulose and laminarin (Fernández-Medina et al. 2022). In brown algal biomass, fucoidan and laminarin are typical polysaccharides constitutive of the cell wall, and if the appropriate enzymes are present, they can release glucose in the hydrolysis stage. Therefore, the cellulose concentration in the seaweed was calculated as the sum of the removable with concentrated acid (ADL) and removable with acid detergent (ADF) fractions, which correspond to the total carbohydrate composition. The enzymatic activities needed to depolymerise laminarin and fucoidan are presumably produced by the metabolism of the fungus *A. awamori* during the SSF. Laminarin is composed of mannitol and glucose, while fucoidan, besides fucose, may contain other sugars, like xylose, arabinose, rhamnose, glucose or galactose, among others. Hence, the use of the total carbohydrate fraction (ADL+ADF) instead of the specific cellulose fraction (ADL) overestimates the maximum theoretical glucose concentration that can be released. Thus, the results obtained were 60.8 % (NP), 74.0 % (5P), 47.8 % (8P) and 50.4 % (12P) where the maximum value of glucose yield corresponds to the samples that were pretreated with *A. awamori* for 5 days (74.0 %). Therefore, even at the best conditions of seaweed processing, glucose yields do not exceed 75% as a consequence of the calculation method used since other polysaccharides, in addition to cellulose, are considered precursors of glucose exclusively, causing a decrease in the calculated glucose yields. In addition, it is also observed that increasing the pretreatment time to 8 or 12 days results in a decrease in glucose yield even compared to the non-pretreated sample. This may be caused by the metabolism of the fungus during pretreatment, which leads to a consumption of hydrolysed sugars, which will be higher the longer the SSF time.

## Discussion

The characterisation of the chemical composition of *R. okamurae* shows that it could be an appropriate biomass waste for reducing sugar production by biological processing. Thus, their lipid content is in the typical range of 2 to 13% of dry weight for macroalgae (Biris-Dorhoi et al., 2020). Brown macroalgae have been widely used to obtain different value-added products, such as pigments, with fucoxanthin the most prominent of them (Garcia-Perez et al., 2022), or polysaccharides, being alginate the major (Li et al. 2021). These compounds are considered in the neutral detergent (NDF) fraction which was the majority in the alga composition. Regarding the leachate characterisation, of the cations with the ability to form insoluble salts with alginate, only Na^+^ was detected. This is probably due to the high salinity of the seaweed. Accumulation of Na^+^ and K^+^ allows for sustaining seaweed in salinity by operating either Na^+^ inclusion and/or exclusion mechanism (Sivakumar and Arunkumar 2009). Concerning the chloride content in leachate, it is not a problem for SSF since its value is of the same order of magnitude as the ones found in salt solutions used to moisture substrates for fungal solid-state fermentation (Yahmed et al. 2017). Finally, the sulphate content was 122 mg/L, probably coming from the cell wall hetero-polysaccharide fucoidan, which is mainly made up of L-fucose sulphated at 2 and 4 positions (Sivakumar and Arunkumar 2009).

In this study, *R. okamurae* has been processed for sugar production by a biological pretreatment followed by an enzymatic hydrolysis step. The results obtained in the biological pretreatment indicated that a certain amount of reducing sugars was released before the inoculation with *A. awamori* spores. Those sugars must have been produced as a consequence of the sterilisation of the seaweed in the autoclave before the biological pretreatment. This unavoidable thermal treatment would have broken the structures of some of the polysaccharides present in the algae, producing monosaccharides. Afterwards, the temporal evolution of TRS along the biological pretreatment is a result of a balance between the reducing sugars released from the hydrolytic activity of the fungal enzymes produced during the SSF process and the parallel sugar consumption by the fungus metabolism. Alginate lyase and FPase activities were measured along the SSF of the seaweed and their secretions patterns related to the cell wall composition. The inner layer of the cell wall of brown algae is mainly composed of cellulose, while the outer one is an amorphous matrix consisting of alginate and fucoidan (Davis et al. 2003). Thus, the presence of alginate in the outer layer probably induced the secretion of alginate lyase. Thus, alginate lyase activity increased significantly after 2 days of SSF and peaked on the fifth day. Once alginate molecules are broken, cellulose fibres are more accessible for FPase attack. After the eighth day of pre-treatment, the enzyme activity declines as a consequence of the depletion of polysaccharide content in the matrix.

Regarding the distribution of the main monosaccharides along the biological pretreatment, it can be pointed out that it is a majority in glucose, mannitol and fucose, as typical for brown algae. In fact, other species of brown seaweeds have already been used as a source of glucose or mannitol for the production of biofuels (Chades et al. 2018) and even recently for the production of lactic acid from fucose (Nagarajan et al. 2022). The variation of the relative percentages of glucose, mannitol and fucose along the pretreatment suggests that other kinds of enzymes different from cellulose and alginate lyases are secreted during *A. awamori* growth as it was previously reported in *Aspergillus* genera (Rodríguez-Jasso et al., 2013).

The results obtained in the complete algal biomass processing (biological pretreatment plus enzymatic hydrolysis) indicate that 5 days is the recommended time of biological pretreatment to obtain a high concentration of glucose in the algal hydrolysates, together with other sugars such as mannitol, arabinose or fucose. It was observed that all the monosaccharides measured through the SSF (Fig. 3) were present in the final extracts from the hydrolysis (Table 3), except xylose, sorbitol and sucrose. It can be explained because their initial percentages after SSF were very low. Thus, during the enzymatic step, mainly glucose was liberated, resulting in a final broth mostly with glucose, with the advantage of having almost only easily fermented sugars.

Pretreatment times up to 8 days lead, probably, to the depletion of polysaccharide content of algal biomass. Therefore, despite the easier access to the internal material of the algal biomass that fungal pre-treatment produces, the depletion of the internal polysaccharides causes the subsequent release of sugars in the hydrolysis step to decrease.

Usually, the processing of brown macroalgae is carried out by a two-step treatment as in this study. However, in most of the studies, the first step consists of an acid pretreatment of the seaweed, and in the second one, it is treated with enzymes. The most common acid employed is sulphuric acid, but cases of pretreatment with hydrochloric acid have also been reported (Ravanal et al. 2017). However, there is a risk of toxin production derived from the toughness of the acid treatment. With the proposed processing in this study, which includes a biological pretreatment of the algal biomass for 5 days, followed by enzymatic saccharification for 24 h with Cellic CTec2®, a TRS yield of 239 g/kg of dried biomass can be obtained, which corresponds to a glucose yield of about 230 g/kg of dried biomass. This process avoids the use of acids in the pretreatment and only uses Cellic CTec2® as an enzymatic agent. This glucose yield is of the same order of magnitude as that reported for *S. latissima* (209 g/kg of dried mass), which was pretreated with 2% (v/v) sulphuric acid and hydrolysed with recombinant alginate lyase H (from *Pseudoalteromonas elyakovii*) plus oligoalginate lyases and Cellic CTec2® (Ravanal et al. 2017). Also, Sharma and Horn (2016) have reported the enzymatic saccharification of *S. latissima* with different alginate lyase and Cellic CTec2® blends, reaching a maximum glucose yield of 225 g/kg of dried biomass at optimum conditions. This study, unlike others in which the hydrolysis step is performed with an enzyme mixture that includes alginate lyases plus cellulases, only uses the cellulase preparation Cellic CTec2®. However, during the biological pretreatment stage, the alginate lyases secreted by the fungus would have broken down the structures of the alginate polymers present in the outer layer of the cell wall of the algae, facilitating the subsequent enzymatic attack of the cellulases on cellulose fibres. In this respect, during the biological pretreatment, FPase and alginate lyase activities were measured, obtaining 0.54 IU of FPase per g of dried biomass on the 8th day of fermentation, while alginate lyase activity peaked on the fifth day (1.6 IU/ g of dried biomass).

In conclusion, the complete processing proposed in this study for obtaining free sugars from *R. okamurae* includes a first stage of a 5-day biological pretreatment followed by a 24-h enzymatic hydrolysis stage. The biological pretreatment is performed by the growth of the fungus *Aspergillus awamori* in solid-state fermentation (SSF) on the previously conditioned and autoclaved algal biomass. This kind of pretreatment is a sustainable alternative to chemical pretreatment as no inhibitors for the subsequent fermentation of sugars are produced, and it is also relatively cheap and environmentally friendly. Also, during the fungal pretreatment, different hydrolytic enzymes (namely, alginate lyase) are secreted to the medium, allowing the depolymerisation of alginate and increasing the accessibility to cellulose in the subsequent stage. The production of alginate lyase by the fungus represents an important cost saving in the overall processing, given the high cost of this enzyme. Indeed, it seems interesting to carry out additional studies to analyse the viability of alginate lyase production by *A. awamori* in SFF.

The second stage consists of the enzymatic hydrolysis of biologically pretreated biomass for 24 h with Cellic CTec2® (112 FP-units/g of biomass). The two-stage process gave a high concentration of total reducing sugars in the hydrolysates: 239 g TRS/kg of dried biomass. This sugar concentration allows the use of these hydrolysates for different fermentation processes, such as the production of bioethanol, acid lactic and polyhydroxyalkanoates. This concentration of reducing sugars per gram of algae is similar to those reported in the literature, as previously commented. However, the titers (g/L) in the hydrolysates are relatively low due to the low initial algal concentration subjected to hydrolysis. It could be highlighted that as no viscosity problems with hydrolysates were detected working below 10 % of algal biomass, it is possible to increase the concentration of reducing sugars in the final extracts by increasing the algal biomass concentration and the enzyme doses.

The main sugars found were glucose (95.8 %) and mannitol (1.5 %), followed by galactose (1.0 %), arabinose (0.9 %) and fucose (0.5 %). These sugars include those produced during hydrolysis with the commercial enzyme cocktail Cellic CTec2®, plus those released during fungal growth in the pretreatment stage, and also a certain amount released as a consequence of the autoclaving of the algal biomass. Increasing the pretreatment time to 8 or more days has a negative effect on net sugar production. The complete biomass processing proposed in this study has been applied to the invasive species *R. okamurae*; however, it could be adapted to other species of brown seaweed with a similar composition.

## Data Availability

All data generated or analysed during this study are included in this published article.
